# Information communication technology accessibility and mental health for older adults during the coronavirus disease in South Korea

**DOI:** 10.3389/fpubh.2023.1126900

**Published:** 2023-09-25

**Authors:** Sujin Park, Weihong Zeng, Pianpian Zhao, Yanke Tong

**Affiliations:** ^1^Jinhe Center for Economic Research, Xi’an Jiaotong University, Xi’an, China; ^2^Technology Management, Economics and Policy Program, College of Engineering, Seoul National University, Seoul, Republic of Korea

**Keywords:** mental health, older adults, ICT accessibility, COVID-19, South Korea

## Abstract

**Introduction:**

As society ages and the digital economy continues to develop, accessibility to information and communication technology (ICT) has emerged as a critical factor influencing the mental health of older adults. Particularly, in the aftermath of the COVID-19 pandemic, the need for non-face-to-face communication has significantly increased older adults’ reliance on ICT for accessibility. This transition from a self-motivated engagement to a more socially passive mode of interaction highlights the importance of creating a digitally inclusive aging society.

**Methods:**

This empirical study used pooled cross-sectional data from the Digital Gap Survey conducted in South Korea in 2018 and 2020. It aimed to analyze the association between ICT accessibility and the mental health of older adults during the COVID-19 pandemic.

**Results:**

A significant positive relationship was found between ICT and mental health among older adults in South Korea. However, this positive association weakened during the COVID-19 period. Furthermore, the analysis revealed heterogeneity among older adults by age, sex, and place of residence, with older females in their 70s living in rural areas experiencing the greatest weakening.

**Discussion:**

These results highlight the need for tailored interventions and support mechanisms for specific demographic groups of older adults. We recommend that the South Korean government implement various policies to facilitate the post-COVID-19 digital landscape. These include initiatives such as ICT-related education programs, development of user-friendly e-government systems, and creation of social media platforms designed to accommodate the needs and preferences of older adults.

## 1. Introduction

South Korea has one of the fastest internet networks in the world, is known as an IT powerhouse, and has internet and smartphone penetration rates of 97.6 and 92.7%, respectively (1, 2). Even older adults aged 60 years and above have a smartphone penetration rate of 80.3% (3), which is one of the highest in the world. Given the high internet and smartphone penetration rates, studies have been conducted in South Korea since the early 2000s, and the relationship between older adults’ information and communication technology (ICT) usage and their satisfaction with life, mental health, and overall quality of life (QOL) has been a subject of continuous discussion (4–7).

In general, older adults do not adapt well to social changes, and face social and psychological isolation. ICT use by older adults has been shown to reduce depression and feelings of loneliness, and increase their self-confidence (8). These findings have prompted governments to recommend and implement policies to facilitate older adults’ access to ICT, such as free training programs, to improve their internet and smartphone proficiency. Thus, efforts have been made to make ICT a part of older adults’ leisure activities. However, previous studies have shown that while ICT use can reduce social isolation, its use to pursue new encounters may increase emotional isolation (8). Furthermore, for older adults residing with their families, the utilization of ICT has demonstrated the potential to impede communication with their family members (9), and for older adults who are already socially affluent and highly educated, social connectivity through ICT may be deemed insignificant (10).

With the emergence of COVID-19 at the end of 2019, ICT has exerted a more direct influence on daily lives, making its access and usage by older adults an even more significant social issue. In the context of the pandemic, older adults’ access to ICT has transcended mere leisure activities, such as personal enjoyment or social interactions, which were observed in the past. Instead, it plays a crucial role in ensuring their survival. The pandemic has introduced a significant shift in the perception and necessity of older adults’ access to ICT. Owing to concerns regarding the spread of the infection, the government has largely transitioned most services from in-person to ICT-driven remote services, and personal social activities have been regulated to promote non-face-to-face interactions. As a result, access to and usage of ICT are no longer solely individual choices, but have become socially driven and essential aspects of daily life. In South Korea, many government services including vaccination appointments and government subsidy applications have moved to contactless channels using ICT, resulting in a significant increase in the use of e-government services. According to the Korea National Information Society Agency’s (NIA) 2022 report on the use of e-government services, approximately 90% of South Koreans use e-government services, with the people aged 60 years and above showing a remarkable 12.2% increase in 2021 compared to 2020 (11).

Despite this growth in older adults’ access to ICT in the post-pandemic period, it has been argued that encouraging them to adopt ICT in response to the pandemic could widen the information divide, disrupt their daily lives, and negatively influence their mental health (12–15). Compared to younger generations, older individuals have lower levels of internet usage, motivation to use, and technological proficiency, resulting in a potential widening of the digital divide, leading to social exclusion and inequality (16–18). Additionally, the widespread adoption of ICT in government services, medical facilities, and delivery apps has caused confusion among older adults, making it difficult for them to cope with new technologies (19). During the pandemic, older adults who were vulnerable to physical impairment and infections were unable to visit medical institutions unless appointments were made through ICT. For those unfamiliar with ICT, accessing public services and making online appointments was difficult (19). Furthermore, for older adults who experience virtual social interactions through ICT, the digital stress and burden suggest that virtual contact may not significantly contribute to their mental well-being (20). Consequently, there has been a shift in perspective regarding access to and utilization of ICT by older adults from the previous encouragement of ICT usage. Some studies have suggested that ICT use in remote social interactions may have a positive and beneficial influence on cognitive abilities and reduce feelings of loneliness and isolation among older adults (21–23). However, contrasting viewpoints argue that ICT-facilitated social interactions in a “contact-free society” may have adverse effects on mental well-being, further widening the digital divide (20, 24, 25).

In an ICT-driven “new normal” society initiated by the pandemic, the integration of various aspects of daily life with ICT will continue. However, this trend can lead to digital inequalities and social exclusion among certain populations, raising concerns about its potential negative influences on mental health. It is essential to continuously study and implement policies to identify suitable alternatives that meet the needs of digitally marginalized communities. Considering these social issues, we conducted a study on the association between older adults’ ICT accessibility and mental health during COVID-19, and proposed policy implications to address these challenges and promote inclusivity.

## 2. Literature review

### 2.1. Mental health of the older adults and associated factors

Mental health encompasses emotional, psychological, and social well-being, and is closely related to life satisfaction, subjective well-being (SWB), and QOL. Previous studies have demonstrated significant associations between mental health and life satisfaction among older adults (26–29). Furthermore, they have shown links between mental and physical health (30) and argued that mental health is a fundamental resource for society’s overall well-being (27). Consequently, mental health in older adults has received considerable attention in the fields of social and health economics.

According to a previous study, the mental health of older adults is associated with several key factors including individual characteristics, health, family dynamics, and social interactions (31–40). Empirical evidence suggests significant variations in the mental health of older adults based on factors such as sex, age, educational attainment, occupation, income level, and residential location (31–34). Additionally, mental health can differ according to physical health (35–37), quality of family relationships (38–40), engagement in social activities, availability of social support, and receipt of pension benefits (41–43).

In recent years, alongside the aforementioned factors, the widespread availability of the internet and increased ownership of smartphones and tablet PCs have established ICT as a critical determinant influencing the mental health, overall well-being, and life satisfaction of older adults.

The study of ICT use among older adults is an ongoing endeavor that has yielded various perspectives. Previous studies have shown that the use of the internet, smartphones, and ICT can increase self-efficacy (44), reduce depressive symptoms (45), and positively influence sense of control (46). Consequently, ICT acts as a medium through which socially isolated and physically frail older adults can engage in alternative activities, alleviate depressive symptoms, and influence overall life satisfaction (8, 44–50). Additionally, previous studies have shown that older adults’ use of ICT and access to digital resources enable them to monitor their physical health more effectively, highlighting the potential of ICT to strengthen mental health and promote physical well-being (51, 52).

However, negative perspectives also exist regarding older adults’ use of ICT. These views suggest that ICT access and utilization may hinder face-to-face communication and family interactions, potentially exacerbating social isolation, loneliness, and depression among older adults (9, 10, 53). Considering these previous studies, it can be concluded that ICT access and utilization among older adults may be beneficial primarily for those who do not cohabit with their families or have limited in-person social interactions owing to physical health issues.

### 2.2. The change of the ICT accessibility due to COVID-19 for older adults

The emergence of coronavirus disease (COVID-19) in 2019 has led to significant social changes. During the post-pandemic period, the concept of a contactless society, often referred to as a non-face-to-face society, gained prominence, leading to the continuous expansion of the scope of ICT. Although COVID-19 has played a role in promoting digitization, there are concerns about exacerbating digital inequalities. In an increasingly digitalized society, people with limited ICT skills are more likely to experience exclusion and marginalization. Digital exclusion and division are particularly pronounced among older adults. Compared to younger age groups, older adults are more likely not to use the internet, and have fewer skills in using digital devices to connect with others (15). Consequently, the accessibility of ICT for older adults belonging to the information-marginalized group has become a topic of great interest in many countries.

During the COVID-19 pandemic, studies on older adults’ ICT use can be divided into two main perspectives. From the perspective of encouraging the use of ICT among older adults during COVID-19, it is argued that ICT plays a crucial role in enabling interactions with distant family members and friends in a contactless society, as well as enhancing older adults’ leisure activities, allowing them to maintain a mentally active life in isolated environments (23). This is positively associated with subjective health, well-being, and mental health (54–56). Furthermore, using ICT to perform personal tasks independently can positively influence older adults’ self-esteem and dignity (57).

However, it should be noted that virtual communication facilitated by ICT may amplify feelings of isolation (20), and increased subjective pleasure and improved well-being during the pandemic were found to be effective only when ICT was used for leisure purposes (56). Therefore, the use of ICT for tasks such as work management and accessing public services may not have a positive association with mental health. This is because of older adults’ negative perceptions of technology and the digital divide. Previous studies have shown that despite the introduction of ICT in various social sectors during the pandemic, older adults experience reluctance and difficulties in social participation through ICT due to concerns about cybercrime, negative technological experiences, and the complexity of technology (19, 57). Furthermore, there are individual differences in ICT skills among older adults willing to use ICT (23), which can lead to digital and information inequalities, exacerbating social inequalities (24, 25, 58).

Considering the comprehensive review of previous studies, it is important to note the ongoing debate and contrasting results regarding ICT accessibility in older adults during the pandemic. Some studies suggest the potential benefits of ICT use for the mental health of older adults, while others emphasize the potential negative associations and the existence of digital inequalities. With the accelerated transition toward an ICT-based digital society, it is crucial to investigate the association between ICT and the well-being of older adults, and further explore how ICT can contribute to their lives. Developing policies that foster older adults’ well-being through ICT has become imperative in light of this transition. This study aimed to investigate the association between ICT accessibility and the mental health of older adults during the COVID-19 pandemic.

## 3. Data source and variable selection

### 3.1. Data source

Samples were selected based on cross-sectional databases from the Digital Gap Survey conducted by the National Information Society Agency (NIA) in 2018 and 2020. The survey’s primary objective was to gather essential data to address the digital divide in South Korea and formulate practical policy recommendations for the future. The survey covered various aspects, including the level of digital information access and demographic and sociological factors. Data were collected annually from September to December.

The survey included a diverse sample of participants, including 7,000 South Korean citizens, 2,200 agricultural and fishing workers, 2,200 individuals with disabilities, 2,200 low-income families, 700 North Korean defectors, and 700 married immigrants, for a total annual survey sample of 15,000 people. For this study, a subset of 30,000 original data entries was used, after excluding 2,800 North Korean defectors and married immigrants. Finally, the study analyzed a sample size of 9,301 after excluding 17,899 responses due to incomplete or erroneous data and individuals under 60 years of age, following the WHO’s definition of older adults.

### 3.2. Empirical strategy and variable selection

In addition to the primary variables of ICT accessibility and COVID-19, several control variables were associated with the mental health of older adults. According to previous studies, these variables include demographic, health-related, family-related, and social factors. Demographic factors include individual characteristics such as age, sex, education, occupation, monthly income, living environment, and living area. Health-related factors refer to physical aspects that affect the general health of older adults (30, 59, 60). Social factors include sociological aspects relevant to older adults such as social capital (61) and pensions, whereas family-related factors may include the presence of household members.

To identify factors that were significantly associated with the mental health of older adults in South Korea, a multiple regression equation was expressed as follows:


$$\begin{array}{c} \mathrm{Mental}\;\mathrm{Health}=\beta_{0}+\beta_{1}ICT+\beta_{2}\mathrm{COVID}:19+\beta_{3}ICT*\mathrm{COVID}:19\\ +\beta_{4}Dem+\beta_{5}Fam+\beta_{6}\mathrm{Health}+\beta_{7}\mathrm{Social}+\mu \end{array}$$

*Mental Health*: as a measure of mental health, we used questions 21–7 from the 2018 and 2020 NIA Digital Gap Surveys. Question 21 assessed mental health and subjective satisfaction in various areas of daily life, including economic conditions, leisure and culture, social activities, interpersonal relationships, family relationships, work, education, and government. Therefore, it was appropriate to measure the respondents’ mental health. The response scale is a 4-point rating scale ranging from 1 (very dissatisfied) to 4 (very satisfied). Higher scores indicated better mental health as reported by the respondents.

*ICT*: ICT accessibility represents the potential of older adults to utilize ICT. ICT accessibility for older adults was measured using six items from the NIA’s Digital Gap Surveys conducted in 2018 and 2020 (Questions 1, 2, and 3). Question 1 consists of two items inquiring about ownership of Desktop and Laptop (1. Do you currently have a desktop or laptop available to you? 1–1 Desktop, 1–2 Laptop). Question 2 comprises three items asking about ownership of Cell Phone, Smart Pad, and Smart Device (2. Please answer whether you have a device available to you, 2–1 Cellphone, 2–2 Smart Pad, 2–3 Smart Device). Lastly, Question 3 is composed of a single item querying whether internet connectivity is available at home (3. Is the internet (including wireless Internet) currently available at your home through computers/laptops, smartphones/smart pads, TVs, and game device?). To simplify the analysis, a scoring system was devised: a score of 1 was assigned if the older adults possessed devices (for Questions 1 and 2) or had internet connectivity (for Question 3), and 0 if they did not. (Note: Question 2–1 for the cell phone ownership part, the responses were divided into 1) smartphone, 2) feature phone, and 3) unpossessed. Both 1) smart phone and 2) feature phone were considered to be possessed and were considered as 1.) Total ICT accessibility scores ranged from 0 to 6 with the score of 1, 2 and 3 are the cutoffs of 25, 50 and 75% quarter, respectively. Participants whose ICT accessibility score is in the top 25% of the distribution are defined as the group with high ICT accessibility which we define as ICT accessibility = 1, and the remaining are categorized as the group with low ICT accessibility which we define as ICT accessibility = 0 (62, 63).

*COVID-19*: COVID-19 is a binary dummy variable COVID-19 emerged toward the end of 2019 and gained widespread global attention through news and media starting in January 2020. Accordingly, “0” was provided for data from 2018, and “1” for data from 2020. The interaction variable between ICT and COVID-19 is denoted as ICT*Covid_19.

*Dem*: Dem represents several demographic and sociological characteristics of older adults. These variables include respondents’ age, sex, education level, occupation, type of residence, residential area, and monthly income. Sex is a binary variable, with “0” for female and “1” for male. The final education level was divided into four categories: 1) below elementary school, 2) middle school, 3) high school, and 4) college or higher. Occupation was categorized as 1) employed, 2) agricultural and fishing workers, 3) unemployed, and 4) other. Residence type was classified into four categories: 1) detached house, 2) apartment, 3) town/multiplex house (villa), and 4) other. Residential area is a binary variable, with “0” for rural areas and “1” for urban areas. The monthly income variable is divided into seven categories: 1) KRW 500,000 or less, 2) KRW 500,000-990,000, 3) KRW 1 million – 1.49 million, 4) KRW 1.5 million – 1.99 million, 5) KRW 2–2.49 million, 6) KRW 2.5–2.99 million, and 7) KRW 3 million or more. Demographic and sociological characteristics were used as the control variables.

*Fam*: Fam represents the living arrangements of older adults. It was categorized as follows: 1) living alone, 2) living with a spouse, 3) living with a spouse and children, 4) living with a spouse, children, and parents, and 5) living with others. The “living with others” category includes cases where older adults reside with relatives such as grandparents, grandchildren, aunts, uncles, and others, excluding spouses, children, and parents.

*Health*: health represents people with disabilities registered by the government. Health is a binary dummy variable, with “0” for respondents who are not registered as disabled by the government, and “1” for those with disabilities officially recognized by the government.

*Social*: social represents the social characteristics of older adults, including social support and guarantee of living. Social support is an independent variable that reflects the respondents’ level of support from online and offline sources. Question 17 assessed the social support. However, there was variation in the number of questions comprising this item across the two survey years. In 2018, the items consisted of 20 questions, whereas in 2020, they consisted of 10 questions. For the convenience of analysis, a weighted approach was employed for the 2018 data, where a 50% weight was assigned to the total score. The response scale for this question was based on a 4-point rating scale, resulting in a score distribution ranging from 10 to 40. Higher scores indicate a greater perceived level of social support received by the respondents. The variable guarantee of living is another independent variable in this study, representing low-income families who receive government support. This variable was analyzed using personal information provided by respondents in the questionnaire. Guarantee of living is a binary dummy variable with “0” for respondents not included in the low-income group, and “1” for respondents in the low-income group, who are supported by the government.

## 4. Statistical analysis

### 4.1. Descriptive analysis

Table 1 presents the definitions and descriptive characteristics of the study variables. The total number of observations is 9,301.

**Table 1:** Definitions and descriptive analysis of variables (*N* = 9,301).

Variable	Definition	Mean(S.D)/Number(%)
Dependent variable		
Mental Health	Subjective mental health satisfaction, a four-point Likert scale	2.45 (0.76)
Independent variable		
ICT Accessibility	ICT accessibility = 0	5,414 (58.21%)
	ICT accessibility = 1	3,887 (41.79%)
COVID-19 outbreak Dummy	0) Non	4,458 (47.93%)
	1) Outbreak	4,843 (52.07%)
Demographic and sociological factors		
Age	Respondents’ age	67.58 (5.75)
Sex	0) Female	4,177 (44.91%)
	1) Male	5,124 (55.09%)
Education	1) Below Elementary school	2,487 (26.74%)
	2) Middle school	2,930 (31.50%)
	3) High school	3,499 (37.62%)
	4) College or more	385 (4.14%)
Occupation	1) Employed	4,179 (44.93%)
	2) Agricultural and fishery workers	3,172 (34.10%)
	3) Unemployed	1,948 (20.94%)
	4) Other	2 (0.02%)
Type of residence	1) Detached house	4,938 (53.09%)
	2) Apartment	3,401 (36.57%)
	3) Town/Multiplex House (Villa)	940 (10.11%)
	4) Other	22 (0.24%)
Residential areas	0) Rural area	2,040 (21.93%)
	1) Urban area	7,261 (78.07%)
Monthly income (KRW)	1) Less than 500,000	317 (3.41%)
	2) 500,000–990,000	1,641 (17.64%)
	3) 1 million - 1.49 million	1,525 (16.40%)
	4) 1.5 million - 1.99 million	1,283 (13.79%)
	5) 2 million - 2.49 million	1,223 (13.15%)
	6) 2.5 million - 2.99 million,	874 (9.40%)
	7) More than 3 million	2,438 (26.21%)
Family		
Living arrangement	1) Living alone	1,777 (19.11%)
	2) Living with spouse	5,245 (56.39%)
	3) Living with spouse and children	1,754 (18.86%)
	4) Living with spouse, children, and parents	53 (0.57%)
	5) Other	472 (5.07%)
Health		
Disabled	0) Non-disabled	7,499 (80.63%)
	1) Disabled	1,802 (19.37%)
Social		
Social support	Respondents’ social support online and offline	25.72 (5.35)
Guarantee of living	0) Non-recipients	8,082 (86.89%)
	1) Recipients	1,219 (13.11%)

The mean score for mental health is 2.45 (S.D = 0.76), indicating a moderate level. In the case of ICT accessibility, it has been found that most older adults have low levels of accessibility (58.21%). For COVID-19, 2018 was indicated as “Non” (47.93%) and 2020 as “Outbreak” (52.07%).

The respondents had a mean age of 67.58 (S.D = 5.75). The sample included a higher proportion of male respondents (55.09%), and the most prevalent level of education was high school graduation (37.62%), while the number of individuals with education beyond the college level was the lowest (4.14%). In terms of occupation, the largest category was employed (44.93%). Regarding residential type, majority of older adults lived in detached houses (53.09%) or apartments (36.57%), with a higher concentration in urban areas (78.07%) than in rural areas (21.93%). In terms of monthly income, the largest group consisted of older adults with a monthly income of three million won or more (26.21%). (About 22,973.78 USD at an exchange rate of 1 USD = 1,305.84 KRW).

In terms of living arrangements, the largest category among older adults was couple households (56.39%). When considering disability and guarantee of living, the majority of older adults were non-disabled (80.63%) and did not receive government support (86.89%). The mean score for social support was 25.72 (S.D = 5.35).

## 5. Empirical analysis

### 5.1. Benchmark model

We examined the association between older adults’ mental health and ICT accessibility during the COVID-19 pandemic using an interaction term for ICT accessibility and COVID-19. The results are presented in Table 2. Model (1) was used to identify factors associated with the mental health of older adults. Model (2) examined the association between ICT accessibility and mental health in older adults, while Model (3) examined the association between COVID-19 and mental health in older adults. Model (4) examined the association between ICT accessibility and mental health of older adults during COVID-19 using an interaction term for ICT accessibility and COVID-19.

**Table 2:** Factors associated with the mental health of older adults in South Korea.

Variables	(1)	(2)	(3)	(4)
ICT		0.108***	0.114***	0.155***
		(0.016)	(0.016)	(0.022)
COVID			−0.076***	−0.045*
			(0.015)	(0.020)
COVID*ICT				−0.074*
				(0.029)
AGE	−0.013***	−0.011***	−0.010***	−0.010***
	(0.001)	(0.001)	(0.001)	(0.001)
Sex (base = Female)				
Male	0.047**	0.043**	0.031*	0.033*
	(0.016)	(0.016)	(0.016)	(0.016)
Education (base = Below Elementary school)				
Middle school	0.066**	0.065**	0.070***	0.067***
	(0.020)	(0.020)	(0.020)	(0.020)
High school	0.095***	0.087***	0.103***	0.100***
	(0.022)	(0.022)	(0.022)	(0.022)
College or more	0.102*	0.082*	0.102*	0.098*
	(0.041)	(0.041)	(0.041)	(0.041)
Occupation (base = Employed)				
Agricultural and fishery workers	−0.036	−0.038	−0.021	−0.020
	(0.022)	(0.022)	(0.022)	(0.022)
Unemployed	−0.083***	−0.082***	−0.077***	−0.077***
	(0.022)	(0.022)	(0.022)	(0.022)
Other	−0.432	−0.427	−0.439	−0.428
	(0.587)	(0.577)	(0.575)	(0.575)
Type of residence (base = Detached house)				
Apartment	0.021	0.020	0.023	0.022
	(0.018)	(0.018)	(0.018)	(0.018)
Town/Multiplex House (Villa)	−0.018	−0.020	−0.012	−0.010
	(0.026)	(0.026)	(0.026)	(0.026)
Other	−0.379*	−0.383*	−0.395*	−0.395*
	(0.164)	(0.161)	(0.158)	(0.156)
Residential areas (base = Rural area)				
Urban area	0.039*	0.034	0.035	0.035
	(0.020)	(0.020)	(0.020)	(0.020)
Monthly income (base = Less than 500,000)				
500,000–990,000	−0.020	−0.026	−0.001	−0.007
	(0.044)	(0.044)	(0.044)	(0.044)
1–1.49 million	−0.051	−0.061	−0.030	−0.036
	(0.046)	(0.046)	(0.046)	(0.046)
1.5–1.99 million	−0.049	−0.062	−0.025	−0.033
	(0.048)	(0.048)	(0.048)	(0.048)
2–2.49 million	−0.019	−0.036	0.007	−0.001
	(0.050)	(0.050)	(0.050)	(0.050)
2.5–2.99 million	−0.014	−0.036	0.012	0.003
	(0.052)	(0.052)	(0.053)	(0.053)
More than 3 million	0.014	−0.012	0.042	0.036
	(0.050)	(0.050)	(0.051)	(0.051)
Living arrangement (base = Living alone)				
Living with spouse	0.045*	0.043*	0.032	0.032
	(0.022)	(0.022)	(0.022)	(0.022)
Living with spouse and children	0.071*	0.055	0.041	0.041
	(0.029)	(0.029)	(0.029)	(0.029)
Living with spouse, children, and parents	−0.020	−0.032	−0.043	−0.041
	(0.093)	(0.092)	(0.092)	(0.092)
Other	0.112**	0.093*	0.081*	0.082*
	(0.038)	(0.038)	(0.038)	(0.038)
Disabled (base = Non-disabled)				
Disabled	−0.527***	−0.528***	−0.509***	−0.510***
	(0.024)	(0.024)	(0.024)	(0.024)
Social Support	0.041***	0.039***	0.039***	0.039***
	(0.001)	(0.001)	(0.001)	(0.001)
Guarantee of living (base = Non-recipients)				
Recipients	−0.240***	−0.238***	−0.210***	−0.212***
	(0.028)	(0.028)	(0.028)	(0.028)
Constant	2.282***	2.193***	2.077***	2.080***
	(0.123)	(0.123)	(0.125)	(0.125)
Observations	9,301	9,301	9,301	9,301
R-squared	0.202	0.206	0.208	0.208
*F*	98.07	97.94	96.11	93.34
F-test for joint significance				
ICT-COVID*ICT				29.31***
COVID-COVID*ICT				16.15***

Robust standard errors in parentheses. ***p < 0.001, **p < 0.01, and *p < 0.05.

The results of Model (1) are as follows: age, sex, education level, occupation, type of residence, residential area, living arrangement, disability, social support, and guarantee of living were associated with the mental health of older adults. Mental health decreased with increasing age ( β = −0.013, *p* = 0.000), and male participants had higher mental health scores ( β = 0.047, *p* = 0.003). In the case of education level, middle school graduates ( β = 0.066, *p* = 0.001), high school graduates ( β = 0.095, *p* = 0.000), and college graduates ( β = 0.102, *p* = 0.014) had better mental health than elementary school graduates. In the case of occupation, unemployed people ( β = −0.083, *p* = 0.000) had lower mental health than the employed. Regarding the type of residence, older adults living in environments other ( β = −0.379, *p* = 0.021) than detached houses, apartments, or town/multiplex houses (villas) had poorer mental health. In terms of residential area, older adults residing in urban areas ( β = 0.039, *p* = 0.046) had better mental health than those living in rural areas. In the case of living arrangements, the mental health of those living with a spouse ( β = 0.045, *p* = 0.039), living with a spouse and children ( β = 0.071, *p* = 0.013), and others ( β = 0.112, *p* = 0.003) were higher than that of older adults living alone. For disability and guarantee of living, non-disabled ( β = −0.527, *p* = 0.000) and non-recipients ( β = −0.240, *p* = 0.000) had better mental health than those with disabilities or recipients of support. Higher levels of social support ( β = 0.041, *p* = 0.000) were associated with better mental health among older adults.

Model (2) showed a positive association between ICT accessibility and mental health among older adults (*β* = 0.108, *p* = 0.000). This suggests that greater ICT accessibility among older adults is associated with improved mental health. The results of model (3) indicated that the mental health of older adults was better before the onset of COVID-19 (*β* = −0.076, *p* = 0.000). This suggests a negative association between COVID-19 and the mental health of older adults, indicating that the pandemic had a detrimental effect on mental health. The results of Model (4) showed that during the COVID-19 period, the positive association between ICT accessibility and mental health weakened compared to the pre-COVID-19 period. The interaction variable was statistically significant (*β* = −0.074, *p* = 0.010). The results showed that older people with high ICT penetration had significantly lower levels of mental health during the COVID-19 period compared to the pre-COVID-19 period (−0.119). After controlling for the influence of COVID-19, ICT showed a statistically significant positive association (0.081) with mental health. However, compared to the pre-COVID-19 period, the positive association between ICT and mental health has weakened. Our findings suggest that more frequent access to ICT among older adults since the onset of COVID-19 has not contributed to improvements in their mental health to the same extent as before the pandemic.

### 5.2. Heterogeneity analysis

Table 3 presents a heterogeneity analysis to examine the differences based on the individual characteristics of older adults. Age and sex are commonly considered when analyzing heterogeneity within an aging population. Additionally, the disparity in digital inequality and subjective well-being across different residential areas has long been a topic of discussion in South Korea. Therefore, the digital gaps between residential areas, particularly during the pandemic, have garnered attention (64–67). The older adults were classified according to age, sex, and residential area to explore potential variations in the findings.

**Table 3:** Difference in gender, age cohort, and residential areas.

Variables	Difference in gender		Difference in age cohort			Difference in residential areas	
	Male	Female	60s	70s	Over 80s	Urban area	Rural area
ICT	0.136***	0.183***	0.150***	0.145**	0.166	0.164***	0.122**
	(0.028)	(0.034)	(0.025)	(0.048)	(0.213)	(0.025)	(0.047)
COVID	−0.043	−0.038	−0.098***	−0.003	0.113	−0.064**	0.021
	(0.027)	(0.030)	(0.024)	(0.038)	(0.096)	(0.022)	(0.042)
COVID*ICT	−0.046	−0.117**	−0.017	−0.146*	−0.346	−0.030	−0.280***
	(0.039)	(0.044)	(0.034)	(0.067)	(0.240)	(0.032)	(0.063)
Observations	5,124	4,177	6,560	2,339	402	7,261	2,040
R-squared	0.228	0.193	0.234	0.158	0.134	0.224	0.184
F-test for joint significance							
ICT-COVID*ICT	14.84***	15.18***	29.94***	4.57**	1.58	33.74***	9.92***
COVID-COVID*ICT	5.85**	9.61***	18.44***	3.27*	1.27	10.66***	12.78***

Robust standard errors in parentheses. ***p < 0.001, **p < 0.01, and *p < 0.05. The rest of the variables in the benchmark model are controlled.

Columns 1–2 in Table 3 depict differences between the sexes. The analysis showed that before COVID-19, ICT accessibility had a positive association with mental health for both older male (*β* = 0.136, *p* = 0.000) and female (*β* = 0.183, *p* = 0.000) adults. However, this positive association weakened for female adults during the COVID-19 period (*β* = −0.117, *p* = 0.008). Previous studies have shown that older female adults had higher depression levels than male adults during the COVID-19 pandemic, and older females with poor subjective health had higher levels of fear of COVID-19 than males (68, 69). Thus, it can be concluded that the accessibility of ICT during the pandemic increased concerns and fears of infection among older female adults, which contributed to a negative influence on mental health.

Columns 3–5 in Table 3 show the differences among age cohorts: individuals in their 60s, 70s, and 80s or above. Before the pandemic, ICT accessibility was positively associated with the mental health of older adults in their 60s (*β* = 0.150, *p* = 0.000) and 70s (*β* = 0.145, *p* = 0.003). However, for those aged 80 and above, the association was not statistically significant. COVID-19 was found to be significantly associated only with the mental health of older adults in their 60s (*β* = −0.098, *p* = 0.000). Those in their 60s reported better mental health before the COVID-19 pandemic. Given that the legal retirement age in South Korea is 65 years, mental health was predicted to be higher before COVID-19, because COVID-19 affected the daily work of older adults in their 60s. The interaction term between COVID-19 and ICT accessibility was statistically significant for those in their 70s ( β = −0.146, *p* = 0.028). Adults over 70s may be less willing to use ICTs than those in their 60s and their skills in using ICTs may be lower than those in their 60s, making them more likely to rely on help from family members when they do use them. Therefore, the passive use of ICT in a contactless society during the pandemic is expected to be negatively associated with mental health.

Columns 6–7 of Table 3 present the differences between different residential areas. Prior to the COVID-19 outbreak, the association between ICT accessibility and mental health of older adults was found to be statistically significant in both urban (*β* = 0.164, *p* = 0.000) and rural areas (*β* = 0.122, *p* = 0.010). The mental health of older adults living in urban areas was found to be better before COVID-19 (*β* = −0.064, *p* = 0.005). Previous studies have shown that urban residents report more COVID-19-related mental health problems than rural residents (70). In South Korea, the response to COVID-19 has been more severe in urban than in rural areas. Considering these facts, a significant negative association between COVID-19 and the mental health of older adults residing in urban areas is anticipated. Regarding the interaction term, the positive association between ICT accessibility and mental health of older adults living in rural areas weakened during COVID-19, and was also statistically significant (*β* = −0.280, *p* = 0.000). This result can be attributed to limited access to ICT and lower frequency of use among older adults living in rural areas. Consequently, despite the possibility that rural areas may have been subjected to less severe activity restrictions during the pandemic, older adults in these areas face greater challenges in using ICT resources. These challenges are expected to exacerbate, especially when engaging in activities such as applying for government subsidies or completing official government tasks that rely heavily on ICT platforms.

### 5.3. Robustness check

As a robustness test, we investigate the life satisfaction and the use of e-government among older adults. Life satisfaction, a component of psychological well-being, is strongly correlated with mental health (26–29) and can be used as a proxy variable for mental health (71). As for ICT accessibility, e-government refers to the ability of citizens, including persons with disabilities, to access basic services without having to visit a physical location. The use of e-government can measure the use of ICT to ensure that government resources are accessible to everyone (11). Therefore, e-government usage is used as a proxy indicator for ICT accessibility (72).

Life satisfaction was measured in 2018 and 2020 using the common question number 22, and E-gov was measured using common question number 10–4. Question 22 measured the respondent’s subjective satisfaction with life and consisted of five items (1. My life is close to the ideal; 2. My life is very good; 3. I am content with my life; 4. I have acquired the important things I want in life so far; 5. If I lived my life again, I would not change anything.) The response scale is a 4-point rating scale ranging from 1 (very dissatisfied) to 4 (very satisfied), resulting in a score distribution ranging from 5 to 20. Higher scores indicated higher life satisfaction. However, there were differences in the response rating scales between 2018 and 2020. In 2018, the response scale was a 7-point rating scale, whereas in 2020, it was rated on a 4-point scale. For the convenience of analysis, the 2018 response scale was converted to a 4-point scale. (Note: The 2018 response scale was converted from a 7-point rating scale to a 4-point rating scale: (1) Strongly Disagree, (2) Disagree, Somewhat Disagree (3) Neither Agree nor Disagree, Somewhat Agree (4) Agree, Strongly Agree.)

Question 10–4 measured the extent to which respondents use public services, such as taxes, public services, and welfare information services, on a PC or mobile device. Responses were split between PC and mobile devices, and both were rated on a 4-point rating scale. For the convenience of analysis, PC and mobile responses were weighted at 50% each and combined. The score distribution ranged from 1(never) to 4 (always), with higher scores indicating that users were more likely to access public services via PC and mobile phones. (Note: Question 10–4 can only be answered by respondents who have used the internet in the last month, thus reducing the sample size.)

Column 1 of Table 4 shows the results of the analysis after replacing the dependent variable with life satisfaction in the benchmark model. The results show that ICT is positively associated with life satisfaction (*β* = 0.681, *p* = 0.000), life satisfaction was higher before COVID-19 (*β* = −1.309, *p* = 0.000), and the interaction term between ICT and COVID-19 is statistically significant for life satisfaction (*β* = −0.273, *p* = 0.007). Column 2 of Table 4 shows the results of the analysis after replacing the existing independent variable ICT with E-gov. The results show that similar to the benchmark model, E-gov has a significant positive association with mental health (*β* = 0.179, *p* = 0.000), while COVID-19 is not significant; however, the interaction term between E-gov and COVID-19 is statistically significant (*β* = −0.054, *p* = 0.050). Column 3 of Table 4 shows the results of the analysis by replacing the dependent variable with life satisfaction and the independent variable with E-gov in the benchmark model. The results show that E-gov (*β* = 0.906, *p* = 0.000) and COVID-19 (*β* = −0.942, *p* = 0.000) were significantly associated with life satisfaction. The interaction term between E-gov and COVID-19 was also statistically significant (*β* = −0.382, *p* = 0.000). The robustness checks show that the interaction terms are statistically significant in all three models (*p* < 0.05). These results, along with those of the benchmark model, demonstrate that ICT accessibility is positively associated with mental health in older adults. However, during the COVID-19 period, the positive association between ICT accessibility and mental health has weakened.

**Table 4:** Robustness check: factors associated with the mental health of older adults in South Korea.

Variables	(1)	(2)	(3)
	Life satisfaction	Mental health	Life satisfaction
ICT	0.681***		
	(0.075)		
E-gov		0.179***	0.906***
		(0.022)	(0.090)
1.COVID	−1.309***	−0.052	−0.942***
	(0.069)	(0.043)	(0.156)
1.COVID#c.ICT	−0.273***		
	(0.100)		
1.COVID#c. E-government		−0.054*	−0.382***
		(0.028)	(0.108)
Constant	8.290***	1.703***	7.691***
	(0.442)	(0.178)	(0.628)
Observations	9,301	6,023	6,028
R-squared/Pseudo R-squared	0.288	0.201	0.281
F-test for joint significance			
ICT-COVID*ICT	52.39***		
COVID-COVID*ICT	361.33***		
E-gov-COVID* E-gov		58.27***	86.16***
COVID-COVID*E-gov		25.09***	261.02***

Robust standard errors in parentheses. ***p < 0.001, **p < 0.01, and *p < 0.05. The rest of the variables in the benchmark model are controlled.

## 6. Conclusions and discussions

This study examined the association between ICT accessibility and mental health in older adults during the pandemic. Multiple regression models were used and heterogeneity and robustness analyses were performed.

During the pandemic, ICT accessibility of older adults maintained a positive association as it did before the pandemic; however, this association weakened during the pandemic. We included the ICT × COVID-19 interaction variables in this study. The results confirmed that when controlling for ICT levels, the positive association between COVID-19 and the mental health of older adults decreased, as supported by the joint significance test. These results suggest that ICT accessibility during the COVID-19 pandemic has a comparatively smaller association with mental health than that before the pandemic, and may even contribute to a decline in mental health.

There are two reasons for this finding. First, when ICT is used for leisure activities or social interaction, it is positively associated with mental health (73–75). Previous studies have shown that ICT is positively associated with mental health when mediated by social support or activities. However, owing to the ubiquity of ICT during the pandemic, older adults without ICT training may feel uncomfortable. This creates a digital divide, in which ICT use becomes socially passive. Previous studies have argued that more frequent ICT use during the pandemic led to discomfort among older adults and created a digital divide (19, 57, 76). Furthermore, older adults use ICT primarily for social interaction, and struggle with internet shopping and financial transactions. Subjective well-being increased significantly when older adults used ICT for leisure purposes. These findings indicate that ICT plays an important role in improving mental health when actively used for social interactions and leisure, while its passive use in online work or shopping may be detrimental to mental health (56, 77, 78). The tasks of public institutions or vaccine applications through ICT during the COVID-19 pandemic have negatively influenced the mental health of older adults. Second, frequent ICT exposure during the pandemic increased anxiety about COVID-19, thereby affecting mental health. Studies have shown anxiety and depression among older adults due to COVID-19 (79–82) and found that anxiety was higher among older adults who received COVID-19 information through social media (83, 84). Thus, emotional contagion on social media can affect mental health.

In Model 1 of the benchmark regression analysis, age, sex, education, occupation, type of residence, residential area, living arrangement, disability, social support, and guarantee of living were associated with the mental health of older adults. These findings support previous studies, indicating that demographic factors in older adults are associated with mental health (31–34). In terms of age, mental health tended to decline as individuals grew older. This could be attributed to older adults experiencing physical vulnerabilities and cognitive decline compared to younger generations, which may amplify feelings of depression. Mental health is expected to deteriorate with age. Additionally, mental health was better among males than females. This finding is consistent with that of a previous study indicating that older females are generally more likely to experience mental health disorders than younger females (32). Furthermore, higher levels of education and employment were associated with better mental health. This could be attributed to individuals with higher education attaining better social positions, and highly educated individuals or those in professional occupations having better prospects for maintaining employment and engaging in social activities even in their later years, leading to better mental health outcomes. With regard to residential status, not residing in apartments, townhouses, or villas was associated with poor mental health. In South Korea, not residing in such a residential environment is often indicative of financial difficulties. Therefore, it can be predicted that individuals residing in “other” types of residences might have the lowest mental health levels among the three categories, excluding apartments, townhouses, and villas. Residing in urban areas and living with family members were associated with better mental health. This could be attributed to urban areas having stronger healthcare systems than rural areas (34), and that living with family members may reduce feelings of loneliness and depressive symptoms among older adults. Furthermore, social support can decrease feelings of loneliness among older adults and enhance their self-confidence and self-esteem, leading to better mental health outcomes. Thus, higher levels of social support are likely associated with better mental health among older adults. This study highlights the importance of emotional factors, such as social support, in the mental health of an aging population (38–43). Finally, disability and guarantee of living were associated with mental health in older adults (85, 86). However, in contrast to a previous study (66), monthly income was not associated with mental health in older adults.

Heterogeneity analysis revealed that during COVID-19, the positive association between ICT accessibility and mental health was most significantly weakened among older female adults in their 70s residing in rural areas. Previous studies have shown that females generally have higher rates of depression and are more vulnerable to external environmental factors (67, 87); female adults in their 70s living in rural areas may have been affected by emotional contagion, such as concerns and fears of infection, while using ICTs during COVID-19. Furthermore, considering the digital divide between rural and urban areas in South Korea, older adults living in rural areas may have had difficulty shopping online or using public services via ICT during the COVID-19 pandemic.

This study proposes a few policy recommendations. First, the South Korean government should implement regular ICT education programs specifically tailored to older adults. These programs should focus on raising awareness about ICT and providing training on using ICT devices and platforms effectively. It is important to address any potential feelings of alienation or discomfort that older adults may have toward ICT, and emphasize the benefits and convenience it can bring to their lives. Given that older adults may have a slower adaptation rate to the digital society than younger age groups, periodic ICT education will greatly assist them in integrating into the digital society. Second, when providing e-government services to older adults, the government should ensure that the systems are user-friendly and easy to understand. Many of South Korea’s subsidy systems and vaccination applications are implemented through e-government platforms that utilize ICT. However, if these systems are complex and difficult for older adults to navigate, they can negatively influence their mental health. Therefore, it is crucial to design and develop user-friendly systems that cater to older adults’ needs and capabilities. Finally, promoting older adults’ participation in social communities facilitated by ICT can improve their mental health. Online gaming companies and local governments operate senior portal sites in South Korea. However, public awareness of these platforms is relatively low. The government should support social media sites, platforms, and communities that specifically target older adults, and encourage their active engagement and participation in society through ICT. The proposed policies aim to address the specific challenges faced by older adults in accessing and utilizing ICT, ultimately promoting their mental health during the digital age.

## 7. Limitations and perspectives

One notable limitation of the present study is its reliance on pooled cross-sectional data, which allows the observation of associations between mental health and ICT among older adults during the COVID-19 pandemic, rather than establishing causal relationships. The absence of panel data restricted our ability to examine temporal dynamics and draw definitive conclusions regarding causality.

Furthermore, the measurement of ICT accessibility was limited to indicators such as ICT ownership and internet access among older adults. Specific purposes of ICT use, such as online shopping, leisure activities, and engagement with e-government services, have not been differentiated. Consequently, this study did not capture the nuanced aspects of ICT accessibility and usage over time, which limited our ability to analyze the specific pathways through which ICT accessibility influences mental health.

However, our study found a weakening positive association between older adults’ access to ICT and mental health during the COVID-19 period. These findings suggest that in a post-pandemic society where ICT adoption is expected to accelerate, our results will contribute to the formulation of ICT-related policies for older adults.

To address these limitations, future studies should employ rigorous methodologies, including panel data analyses, to investigate the causal relationship between ICT accessibility and mental health outcomes in older adults. Additionally, it is essential to expand data collection efforts to encompass specific domains of ICT use, such as social interaction, e-government engagement, and online shopping. This will provide a more comprehensive understanding of the potential influence of ICT on mental health, and facilitate the development of targeted policy proposals tailored to the specific contexts of social interaction, e-government use, and online shopping among older adults.

## Data availability statement

The original contributions presented in the study are included in the article/supplementary material, further inquiries can be directed to the corresponding author.

## Author contributions

SP conceived the idea and collected the original data. WZ participates the research design, empirical strategy, and gave comments on the draft and polished it. SP, PZ and YT worked in data cleaning and statistical analysis. SP and PZ drafted the manuscript and edited the paper. All authors contributed to the article and approved the submitted version.
